# Effects of a Remote Antimicrobial Stewardship Program on Antimicrobial Use in a Regional Hospital System

**DOI:** 10.3390/pharmacy8010041

**Published:** 2020-03-16

**Authors:** Joshua Knight, Jessica Michal, Stephanie Milliken, Jenna Swindler

**Affiliations:** McLeod Health, 555 East Cheves Street, Florence, SC 29506, USA; joshkni@gmail.com (J.K.); stephanie.milliken@mcleodhealth.org (S.M.); jenswindler@mcleodhealth.org (J.S.)

**Keywords:** antimicrobial stewardship, antibiotic utilization, stewardship interventions, pharmacist

## Abstract

While antimicrobial stewardship programs (ASPs) are well established at most large medical centers, small or rural facilities often do not have the same resources; therefore, different methods must be developed to start or expand ASPs for these hospitals. The purpose of this quality improvement study was to describe the implementation of a pharmacist-led remote ASP and assess the effect on antimicrobial use. Antimicrobial use in days of therapy per 1000 patient days (DOT/1000 PD) was compared between the six months before and after remote ASP implementation. Changes in system-wide, facility-specific, and target antimicrobial use were evaluated. Pharmacist interventions, acceptance rates, and number of times infectious disease (ID) physician assistance was sought were also tracked. System-wide antimicrobial use was 4.6% less in the post-implementation time period than in the pre-implementation time period, with vancomycin, piperacillin/tazobactam, and fluoroquinolones having the greatest reductions in use. Ninety-one percent of interventions made during the post-implementation period were accepted. ID physician review was requested 38 times, and direct ID physician intervention was required six times. Remote ASPs delivered from a central facility to serve a larger system may reduce antimicrobial use, especially against targeted agents, with minimal increase in ID physician workload.

## 1. Introduction

Antimicrobial stewardship is defined as the systematic effort to increase appropriate use of antimicrobials to help improve patient outcomes, reduce antimicrobial resistance, and reduce cost [1]. Responsible use of antibiotics is of extreme importance, especially for institutions with limited access to newer agents with activity against multidrug resistant pathogens. In a 2012 consensus statement, the Society for Healthcare Epidemiology of America, the Infectious Diseases Society of America (IDSA), and the Pediatric Infectious Diseases Society uniformly recommended implementing antimicrobial stewardship programs (ASPs) throughout the healthcare system [2]. In 2016, IDSA released guidelines for implementing and maintaining an ASP [3]. In 2019, implementation of an ASP became a condition of participation for the Centers for Medicare and Medicaid Services [4].

ASPs have been shown to effectively decrease inappropriate use of antimicrobials, antimicrobial resistance, and the occurrence of *Clostridioides difficile* infection, a common and serious adverse effect of antibiotic use [5]. In 2017, Libertin et al. examined the effects of ASP initiation at a rural community hospital and found a statistically significant decrease in antimicrobial use, measured in days of therapy per 1000 patient days (DOT/1000 PD) [6]. Additionally, Baur et al. published findings that ASP implementation significantly reduced the incidence of extended-spectrum beta-lactamase-producing organisms, methicillin-resistant *Staphylococcus aureus* (MRSA), and *C. difficile* infections [7]. Similarly, a 2014 meta-analysis found that ASPs significantly decreased *C. difficile* rates (risk ratio 0.48, CI 0.38–0.62) [8]. In addition to increasing responsible use of antimicrobials and slowing resistance development, ASPs positively affect patient outcomes. In 2014, a retrospective cohort looking at adults with skin and soft tissue infections found that interventions made by an ASP pharmacist decreased length of stay (4.4 versus 6.2 days, *p* < 0.0001) and 30 day all-cause readmission rates (6.5% versus 16.71%, *p* = 0.05) compared to historical data in patients with the same disease states [9]. Furthermore, a meta-analysis published in 2016 found that guideline-adherent medical therapy reduced patient mortality (relative risk 0.65, CI−0.54–0.80) [10]. 

Given the literature, national society endorsements, and governmental guidelines espousing their implementation, ASPs have become increasingly common in hospitals around the United States. Although an ASP has been established at McLeod Health’s central facility since 2010, smaller hospitals within the health system have lacked formal programs. In October 2018, antimicrobial stewardship services were expanded to cover all locations. As part of this expansion, ASP pharmacists began performing remote antimicrobial stewardship from the central facility. The present study describes that expansion, including intervention acceptance rates and antimicrobial use trends during the six months before and after program expansion.

## 2. Materials and Methods 

### 2.1. Study Setting

McLeod Health is a seven-hospital system with a total bed count of 965. The flagship hospital of the institution, McLeod Regional Medical Center, is a 461-bed acute care, community hospital with infectious disease (ID) physician consult services consisting of two full-time equivalent (FTE) physicians. The remaining six hospitals serve lower acuity patients, do not have in-house ID physicians, and differ significantly in size and pharmacy services offered (Table 1). In addition, the health system has two microbiology laboratories open seven days a week. One, located at the flagship hospital, provides services to five facilities including the facility excluded from this study, while the second provides services to two. One of the seven hospitals was excluded from the study due to using a different electronic medical record (EMR) that could not calculate DOT data. 

Of the facilities for which ASP services began in October 2018, none had decentralized clinical pharmacist services. Two of these (facilities C and E) had Monday through Friday discharge planning rounds which pharmacists could attend with the hospitalists and case managers. All hospitals had pharmacist protocols for renally adjusting antibiotics, transitioning parenteral agents to oral, and pharmacokinetic dosing for vancomycin and aminoglycosides. Protocols were identical for each facility. Only the flagship hospital has a policy and resources for penicillin skin testing. No order sets were changed related to ASP expansion during the study period.

ASP pharmacists were allowed per protocol to order repeat blood cultures for patients with *S. aureus* bacteremia or fungemia as well as monitor creatine phosphokinase (CPK) for patients receiving daptomycin.

### 2.2. Preparation 

An ASP subcommittee was created that reported to a system-wide Pharmacy and Therapeutics committee. The ASP subcommittee was chaired by an ID physician and led by two ASP pharmacists. Other members included non-ID physicians, clinical microbiologists, pharmacy directors from each facility, infection preventionists, and nurses. To assist with buy in, at least one physician was chosen from each facility to champion antimicrobial stewardship efforts and participate in the ASP subcommittee. In preparation for remote ASP implementation, ASP pharmacist positions were increased from one half FTE to two full FTEs. 

Meaningful metrics were decided upon prior to starting remote ASP. These included system-wide total antimicrobial use measured as DOT/1000 PD, facility-specific total antimicrobial use, and use of target antimicrobials. Target formulary antimicrobials were chosen based on goals to reduce DOTs for broad-spectrum antimicrobials (cefepime, piperacillin/tazobactam), fluoroquinolones (ciprofloxacin, levofloxacin), carbapenems (ertapenem, meropenem), and clindamycin; and to monitor Gram-positive agent use (vancomycin, linezolid, daptomycin, ceftaroline). Pharmacist interventions were also tracked this way, with total interventions and interventions per 1000 patient days on system-wide and facility-specific levels. ID physician assistance was also tracked to assess workload for these providers. This information was reported monthly for all facilities and to all facilities after the remote ASP went live.

### 2.3. Implementation 

Beginning in October 2018, two ASP pharmacists provided antimicrobial stewardship services system-wide, including on-site visits to every site once monthly. Each on-site visit lasted a minimum of eight hours to allow for provider interaction. Each pharmacist was given a company laptop and wireless phone to perform their jobs. The pharmacists used their personal vehicles to travel to each site; however, they were provided stipends according to the number of miles driven. Included facilities utilized the same clinical decision support software that allowed the ASP pharmacists to proactively intervene. The excluded facility was provided the contact information for the ASP pharmacists for questions as needed since the pharmacists did not have access to that facility’s EMR. 

ASP pharmacist services were available Monday through Friday from 07:30 to 18:00 and consisted of primarily prospective audit and feedback including reviews of cultures; antimicrobial length of therapy specifically targeting patients exposed to at least seven days of therapy; targeted antimicrobials including antiretroviral agents; and dual anaerobic, beta lactam, atypical, or MRSA therapy. ASP pharmacists made recommendations directly to the involved provider or indirectly through on-site pharmacists. Interventions were documented in the EMR at the request of the provider, otherwise they were only documented in the clinical decision support software. For non-flagship facilities, the majority of non-protocolized interventions were made via direct phone call to physicians. At the flagship facility, interventions were more often made through decentralized pharmacists. An ID physician was available for additional patient review or direct intervention as needed.

The ASP pharmacists initially divided daily patient care responsibilities into one pharmacist covering all remote sites while the other covered the flagship hospital, rotating every one to two weeks; however, this evolved into each pharmacist covering half of the flagship hospital on a non-rotating basis plus two of the non-flagship facilities rotating monthly. Facility D mainly consisted of long-term acute care patients and was covered by one ASP pharmacist on a non-rotating basis. ASP pharmacists spent 50% to 75% of time each day reviewing non-flagship facilities; however, given the increase in ASP pharmacist FTEs, the net amount of ASP review at the flagship hospital was largely unchanged compared to the pre-implementation period.

### 2.4. Study Design

This quality improvement project utilized a quasi-experimental, pre–post study design and was exempt from institutional review board oversight. Baseline antimicrobial use was established using data from April to September 2018, while post-implementation data were collected from October 2018 to March 2019. DOT/1000 PD was calculated manually by dividing antimicrobial DOT by patient days as provided by the McLeod Health finance department and multiplying by 1000. Antimicrobial DOTs were defined as the administration of a specific medication on a calendar day and were collected through TheraDoc™ clinical surveillance system for all included facilities for all months except March 2019. Two of the included facilities (Facilities B and E) changed EMRs that month, and so their DOT data were generated through Cerner Millennium^®^. Patient days were collected from the McLeod Health finance department and included inpatient adult, inpatient pediatric, inpatient neonatal, and swing bed days. Behavioral health and Hospice patient days were not included due to almost no antimicrobial use from those areas. Patient days are not calculated for emergency department beds, and so emergency department stays did not contribute to the denominator.

Pharmacist interventions were reported directly from the ASP pharmacists. Patient-level data was not generated. 

### 2.5. Outcome Measures

The primary outcome was system-wide antimicrobial use in total antimicrobial DOT/1000 PD before and after implementing a remote ASP. Secondary outcomes included individual facility total antimicrobial use and individual facility and system-wide use of target antimicrobials (carbapenems, fluoroquinolones, clindamycin, vancomycin, linezolid, daptomycin, ceftaroline, piperacillin/tazobactam, and cefepime). Antibiotic use was measured in DOT and standardized per 1000 patient days. Provider acceptance rate of ASP pharmacist interventions and need for ID physician assistance was also assessed. Intervention acceptance rate was tracked as both total interventions and interventions per 1000 patient days at individual facilities to help account for significant differences in facility sizes. ID physician assistance was tallied by ASP pharmacists who tracked whether the ID physician reviewed the chart and provided recommendations through the ASP pharmacist or had to contact the involved provider directly. ASP pharmacist recommendations for formal ID consults were tallied separately.

### 2.6. Statistical Analysis

Descriptive statistics were calculated using Microsoft Excel^®^ 2013, Microsoft Inc. χ² was performed on days of antibiotic therapy pre- and post-intervention using Stata^®^ version 14.2 (StataCorp LP, College Station, TX, USA).

## 3. Results

**Figure 1:**
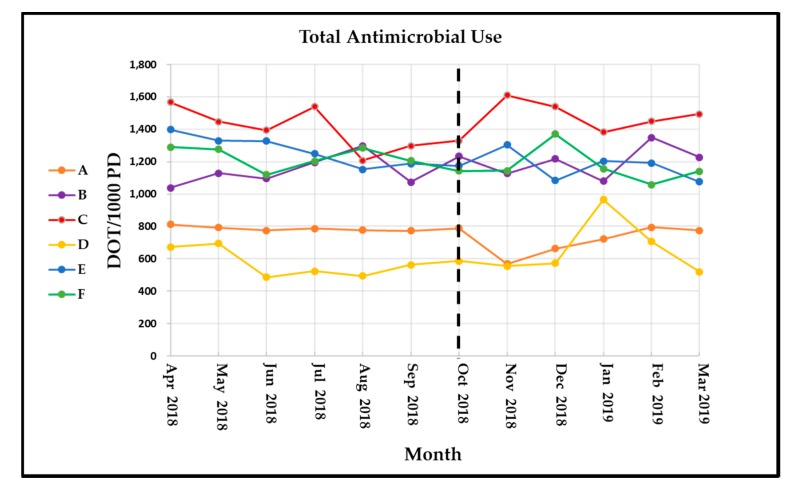
Monthly, facility-specific, total antimicrobial use measured as days of therapy per 1000 patient days (DOT/1000 PD). Dashed line indicates time at which the antimicrobial stewardship program expanded to all facilities in the health system.

System-wide antimicrobial use was 4.6% lower during the six months after implementing the system-wide ASP compared to baseline (Table 2). Of the tracked antimicrobials, the largest overall DOT reductions were observed with vancomycin and fluoroquinolones. Non-flagship facility antimicrobial use data is summarized in Table 3 and shows a 2% increase in total antimicrobial use in the post-implementation period that was statistically significant. Monthly facility-specific total antimicrobial use is displayed in Figure 1. Ninety-one percent (1581 of 1738) of ASP pharmacist interventions were accepted by providers (Table 4 and Table 5). The system-level ASP pharmacist intervention rate was 107 interventions per 1000 patient days. An ID physician review was requested 38 times, and direct ID physician intervention was required six times during the post-implementation period.

## 4. Discussion

Utilizing already established ASPs at a central facility to remotely provide services to smaller facilities within the same health system is not a new concept. A report by Wood and colleagues published in 2015 describes a remote, pharmacist-driven ASP for six community hospitals within one health system that reports similar results with favorable physician acceptance rates (83%–89%) and reductions in target antimicrobial utilization with no significant decrease in total antimicrobial use [11]. A different remote ASP model described by Shively and colleagues wherein ID physicians provided antimicrobial stewardship services via telehealth to two community hospitals also reported similar acceptance rates (88.9%); however, they found statistically significant reductions in antimicrobial use (342.1 vs. 258.7 DOT/1000 PD, *p* < 0.001) [12].

While our system-wide data shows positive trends during the six months after implementing remote ASP services, this was primarily driven by the flagship facility. System-wide, total antimicrobial use was chosen as our primary outcome to assess global trends and establish system-wide goals to stabilize fluctuations related to intra-system patient transfers and varying percentages of swing beds per facility that skew antimicrobial use metrics at individual facilities. Facility-specific data were assessed primarily to identify site-specific antimicrobial trends to develop targets for interventions. 

Overall antimicrobial use increased slightly in the post-implementation period for non-flagship facilities. Examining only target antimicrobials, antibiotic use decreased by 9.3 DOT/1000 PD in the post-implementation phase compared to the pre-implementation phase, meaning that non-target or narrow-spectrum antimicrobial use increased by 32 DOT/1000 PD. The most significant reduction for non-flagship facilities was with meropenem use. Meropenem overuse was a primary driver for our ASP expansion, thus one of the most highly targeted agents for interventions.

Vancomycin and fluoroquinolones were the main drivers of antimicrobial use reduction. Reductions in antimicrobial use were more pronounced at smaller facilities with much larger month-to-month fluctuations. This effect was even more pronounced with broader-spectrum agents that were rarely used. This was expected, since an ASP was already established at the central facility. Not every target antimicrobial saw use decreased in the post-implementation time period, which may be at least partially explained by increased appropriate use of those agents. For example, ertapenem use increased in the post-implementation period while meropenem use decreased. This may reflect appropriate switches from meropenem to ertapenem to spare anti-Pseudomonal coverage in resistant Enterobacteriaceae infections.

It is important to note that, with the ASP in its nascence, a primary goal for our ASP pharmacists was relationship building with providers. One of the barriers to providing remote ASP services is that face-to-face contact between the ASP pharmacists and providers is limited. Our data show that ASP pharmacists can effectively provide remote ASP services, even at facilities where relationships between providers and de-centralized clinical pharmacists are not well established. That being said, the ASP pharmacists reported being less aggressive with recommendations and focusing on high-impact interventions with a high likelihood of success. It is likely that this approach also contributed to the low rejection rate for ASP pharmacist interventions.

The majority of rejected interventions involved either excessive length of therapy or excessive antimicrobial spectrum. ID physician assistance was primarily employed when an ASP pharmacist recommendation was rejected and there was concern for patient harm as a result or in cases for which a diagnostician was required, often related to *S. aureus* bacteremia.

Methods for tracking antimicrobial use vary. There are numerous metrics used to track antimicrobial use and ASP efficacy, but there is no clear consensus as to which metric is preferred [13]. DOT/1000 PD was chosen for this study because, compared to other metrics, DOT offers the best balance of feasibility and applicability. Furthermore, the addition of patient days to the denominator helps to compare facilities of different sizes. Other common ASP metrics include defined daily dose (DDD), cost of therapy (COT), and length of therapy (LOT). DDD compares the amount of drug used against the average maintenance dose for an antimicrobial’s primary indication. COT may be useful from an administrative perspective, but it fails to capture the clinical significance of certain ASP interventions; there are times when the more expensive drug is the more clinically appropriate choice [14]. LOT, which represents the number of days that a patient receives systemic antimicrobial agents regardless of the number of agents used on any given day, will always be lower than or equal to DOT. While this can be a clinically significant measurement, it often proves difficult to assess due to changes in antimicrobial therapy or treatment of multiple infectious diseases [10,15].

Even though use of patient days in the stewardship metric denominator did help standardize values between facilities, DOT/1000 PD still falls short when comparing facilities of significantly different sizes. This metric overestimates use at smaller facilities, especially when a given facility had fewer than 1000 patient days per month. Given this, establishment of baseline values and monitoring trends proved a more valuable use of DOT/1000 PD than actual interfacility comparison.

It is important to note that conclusions about appropriate use cannot be drawn from the endpoints assessed in this study. Ideally, one of the primary goals of an ASP would be to ensure antibiotic use is appropriate; however, in the early stages of development, ASPs often lack the personnel and resources to systematically assess and record appropriateness of therapy. Instead, resources for new ASPs can be effectively spent targeting broad-reaching, practical initiatives, including standardizing formularies and microbiology laboratory reporting between facilities and assuring order sets align with guideline recommendations. Interventions can also be targeted to complicated disease states associated with high rates of morbidity and mortality (e.g., *S. aureus* bacteremia).

This study has several limitations. The pre- and post-implementation periods span different seasons, which likely skews antimicrobial use results. These time frames were a convenience sample, in that prior data was unavailable. That being said, the post-implementation period included winter months when antimicrobial use would be anticipated to be higher than summer months, and so we hypothesize that antimicrobial use would have been higher than 2% more than summer months if the ASP had not been present at the non-flagship facilities. Additionally, this study did not control for other factors that may have affected antimicrobial use. Rates of *S. aureus* nasal screenings increased in October 2018 at the flagship facility after intensivist, nursing, and pharmacist education to de-escalate vancomycin use in patients diagnosed with pneumonia. While no changes were made to protocols or order sets, anecdotal evidence suggests that this directly decreased vancomycin use at that facility. While this was an ASP initiative, it was not directly related to ASP expansion efforts. Finally, facilities B and E changed EMRs during the last month of the post-implementation period, making it possible that differences seen in antimicrobial use for March 2019 were related to differences in data generation rather than true changes in antimicrobial use for these two facilities.

## 5. Conclusions

This study sought to describe an approach to ASP expansion in one health system and demonstrates that ASPs delivered from a central facility to serve a larger health system may reduce antimicrobial use. While overall change in use was modest, provider acceptance rates for ASP pharmacist interventions were high and workload was not substantially increased for the ID consult team.

## Figures and Tables

**Table 1:** Study facilities.

Facility Code	Bed Count	Average Inpatient Length of Stay, Days	Pharmacy Services	Pharmacy Service Hours	Microbiology Lab Services
A	461	5.7	Decentralized	24 h	On site
B	105	4.4	Centralized	Non-24 h	Remote from facility E
C	79	3.6	Centralized	Non-24 h	Remote from facility A
D	49	22.4	Remote only ^1^	24 h, remote	Remote from facility A
E	50	4.0	Centralized	Non-24 h	On site
F	59	5.2	Centralized	Non-24 h	Remote from facility A

^1^ Services provided by Facility A.

**Table 2:** System-wide antimicrobial use in days of therapy per 1000 patient days before and after implementing a remote antimicrobial stewardship program.

Antimicrobial	Pre-Implementation Period (April 2018–September 2018)	Post-Implementation Period (October 2018–March 2019)	Change in Use from Pre-Implementation to Post-Implementation Period (% Change)	*p* Value
Total antimicrobials	880.6	839.8	−40.8 (−4.6)	<0.001
Anti-Pseudomonal beta-lactam agents				
Piperacillin/tazobactam	131.9	120.8	−11.1 (−8.4)	<0.001
Cefepime	78.9	81.8	+2.9 (+3.6)	0.026
Carbapenems				
Meropenem	15.2	10.0	−5.2 (−34.1)	<0.001
Ertapenem	3.0	3.8	+0.8 (+26.1)	0.003
Fluoroquinolones	73.8	63.7	−10.1 (−13.7)	<0.001
Anti-MRSA agents				
Vancomycin	106.7	90.9	−15.8 (−14.9)	<0.001
Clindamycin	29.0	24.3	−4.7 (−16.1)	<0.001
Linezolid	10.0	11.7	+1.7 (+16.7)	<0.001
Daptomycin	6.7	7.0	+0.3 (+3.7)	0.515
Ceftaroline	3.2	1.6	−1.6 (−49.0)	<0.001

MRSA: methicillin-resistant *Staphylococcus aureus*.

**Table 3:** Antimicrobial use in days of therapy per 1000 patient days before and after implementing a remote antimicrobial stewardship program at non-flagship facilities B, C, D, E, and F.

Antimicrobial	Pre-Implementation Period (April 2018–September 2018)	Post-Implementation Period (October 2018–March 2019)	Change in Use from Pre-Implementation to Post-Implementation Period (% Change)	*p* Value
Total antimicrobials	1152.8	1175.5	+22.7 (+2.0)	<0.001
Anti-Pseudomonal beta-lactams				
Piperacillin/tazobactam	174.8	176.6	+1.8 (+1.0)	0.600
Cefepime	83.6	97.8	+14.2 (+17.0)	<0.001
Carbapenems				
Meropenem	34.4	17.5	−16.9 (−49.1)	<0.001
Ertapenem	2.2	7.4	+5.2 (+236.4)	<0.001
Fluoroquinolones	118.9	118.1	−0.8 (−0.67)	0.799
Anti-MRSA agents				
Vancomycin	158.1	150.5	−7.6 (−4.8)	0.021
Clindamycin	35.4	30.2	−5.2 (−14.7)	0.001
Linezolid	13.4	16.5	+3.1 (+23.1)	0.005
Daptomycin	8.9	8.2	−0.7 (−7.9)	0.447
Ceftaroline	2.5	0.1	−2.4 (−96.0)	<0.001

MRSA: methicillin-resistant *Staphylococcus aureus*.

**Table 4:** Most common interventions by antimicrobial stewardship pharmacists at facility A.

Intervention Type	Post-Implementation Period (October 2018–March 2019)	Acceptance Rate
Laboratory monitoring	79	94%
Antibiotic optimization	78	97%
Stop date determination	73	99%
Antibiotic de-escalation	56	79%
Antibiotic escalation	51	92%
Antibiotic discontinuation	48	90%
ID consult recommendation	22	73%
Outpatient or ED intervention	21	100%
Antibiotic allergy clarification	21	-
Antiretroviral management	21	95%

**Table 5:** Most common interventions by antimicrobial stewardship pharmacists at facilities B, C, D, E, and F.

Intervention Type	Post-Implementation Period (October 2018–March 2019)	Acceptance Rate
Antibiotic discontinuation	83	67%
Antibiotic de-escalation	79	58%
Stop date determination	67	88%
Antibiotic optimization	54	98%
Laboratory monitoring	53	91%
Antibiotic allergy clarification	36	-
Antibiotic escalation	22	95%
Outpatient or ED intervention	16	94%
Antiretroviral management	4	100%
ID consult recommendation	3	66%
